# Developing intensive, short-term courses for public health—Creation and outcome evaluation of the Skills for Health and Research Professionals (SHARP) program at the Columbia University Mailman School of Public Health

**DOI:** 10.3389/fpubh.2024.1467002

**Published:** 2024-10-31

**Authors:** Abigail L. Welbourn, Kasey J. M. Brennan, Andrea A. Baccarelli

**Affiliations:** Harvard T.H. Chan School of Public Health, Boston, MA, United States

**Keywords:** epidemiology, continuing education, professional education, workshop, program evaluation

## Abstract

Short, intensive education programs provide an under-utilized avenue for public health professionals to learn and apply the latest methods and technologies. We report on the creation and implementation of the Skills for Health and Research Professionals (SHARP) program at the Columbia University Mailman School of Public Health. The self-sustaining, concise, intense educational format equips participants with concrete skills, better enabling them to respond to complex public health challenges.

## 1 Introduction

In today's fast-paced world, short, intensive public health education programs are increasingly crucial. The public health landscape continually evolves, presenting new challenges regularly along with new methods and technologies developed to address them. It is crucial for public health professionals to learn and apply these new methods effectively to address emerging challenges and improve public health outcomes (1).

While most professionals are aware of the importance of staying up to date on emerging public health challenges and new research methods, the pace of change can make it difficult to keep up with new practices and approaches. Professionals already juggling multiple responsibilities may be challenged to find the time and resources to upgrade their education. Short, intensive courses provide an opportunity to stay on top of the latest methods and technologies, gain new skills, and apply them efficiently and effectively (2).

Opportunities for training and skill development are increasing (3)—notably massive open online courses (MOOCs) offer a diverse array of learning opportunities (4, 5)—but often lack real-time instructor and participant interaction, are too long or cumbersome to complete, or do not cover specific areas of interest (6, 7). Short, highly targeted, and interactive workshops fill a gap in the educational environment.

## 2 Pedagogical design

We detail here the design, creation, and implementation of the Skills for Health and Research Professionals (SHARP) program at the Columbia University Mailman School of Public Health. The program features bootcamps and workshops led by field experts and delivered in an intensive 2–3-day format, allowing participants to gain new knowledge and skills quickly and efficiently. The SHARP program provides a blueprint for skill-based education and training that enables public health professionals to keep pace with the emerging challenges and latest developments in their field to improve public health outcomes.

SHARP covers four core training sectors: (1) Omics, (2) Data Science, (3) Environment and Climate, and (4) Professional Development (Table 1). Trainings typically provide 16–24 h of intensive theory and hands-on application over 2–3 days, with scheduled networking opportunities for instructors and attendees.

**Table 1:** Columbia University Mailman School of Public Health Current SHARP training offerings.

**Omics**	**Epigenetics Boot Camp: Planning and Analyzing DNA Methylation Studies**
	The Exposome Boot Camp: Measuring Exposures on an Omic Scale
	Mendelian Randomization Boot Camp: A Practical Guide to Study Design and Implementation
	Microbiome Data Analytics Boot Camp: Planning, Generating, and Analyzing 16S rRNA Gene Sequencing Surveys
	Multi-omics Boot Camp: Analysis of Omics Data for Research Studies
	Quantitative Genomics Training: Methods and Tools for Whole-genome and Transcriptome Analyses
	Single Cell Analysis Boot Camp: Systems Biology Methods for Analysis of Single Cell RNA-Seq
Data Science	Causal Mediation Analysis Training: Methods and Applications Using Health Data
	Code Rigor and Reproducibility with R Boot Camp: Design Principles and Practical Tools to Make Research Code More Efficient, Less Buggy, Easier to Reproduce, and Ready to Share
	Electronic Medical Records Boot Camp: Biostatistical Methods for Analyzing EMR Data
	Machine Learning Boot Camp: Analyzing Biomedical and Health Data
	Python Data Wrangling Boot Camp: Introduction to Data Wrangling, Cleaning and Manipulation with the Python Programming Language
	Shiny Boot Camp: Building Interactive Graphics and Dashboards in R
	SQL Boot Camp: Building and Querying Databases
	Statistical Analysis with Missing Data Workshop: Methods and Applications in Health Studies
Environment and Climate	Bayesian Modeling for Environmental Health Workshop: Concepts and Computational Tools for Spatial, Temporal, and Spatiotemporal Modeling Relevant to Public Health
	Climate Change and Health Boot Camp: Building Skills and Knowledge for Effective Engagement
	Environmental Justice Boot Camp: Theory and Methods to Study Environmental Health Disparities
	Environmental Mixtures Workshop: Applications in Environmental Health Studies
	Exposure Modeling Boot Camp: Traditional and Machine Learning Methods for Environmental Epidemiology
	Google Earth Engine Boot Camp: Methods for Using Satellite and Geospatial Data for Environmental Exposure Science
	Life Cycle Assessment Boot Camp: LCA for the Health Sector
	GIS Workshop: Visualizing and Analyzing Health Data
	Indigenous Environmental Health Research Workshop: Methods, Ethics and Practice to Collaborate with Communities
	Radiation Safety Officer Training
Professional Development	Creating Compelling Research Narrative Workshop: Strategies for Logically Presenting Your Science
	NIH Grant Writing Boot Camp: Building a Strong Foundation for Funding Success
	PI Crash Course: Skills for Future or New Lab Leaders
	The PI's Business of Research Boot Camp: Ins and Outs of Budgets, Personnel and Project Management

Workshop formats and teaching styles are tailored to best suit the topic area. All attendees are expected to complete workshops with concrete skills that they can immediately apply to their area of interest. Hands-on activities are incorporated into most workshops and instructors provide real-time guidance on trainee work. Participants who attend and participate in all workshop modules are provided with a certificate of completion at the conclusion of the trainings.

## 3 Learning environment and objectives

In 2017, we designed a 2-day hands-on epigenetics boot camp working with real methylation data to address the shortage of epigenetic training in public health. While it had become clear to basic science programs that epigenetics were important drivers of health, the topic was rarely part of formal public health curricula, leading to a shortage of researchers capable of conducting large-scale epidemiological studies of epigenetics. There was a gap in the educational offerings: plenty of learning opportunities covered epigenetics, but few resources were available to help researchers apply the new analysis technologies to their epidemiology studies. The reams of data produced by epigenetic analyses were an additional stumbling block, as few researchers had both the biological understanding of what the data meant and the computational skills to analyze statistically complex datasets. Our boot camp bridged that gap by providing both an overview of what was possible to analyze with the current technologies and the skills needed to interpret the produced data.

The success of the initial boot camp highlighted the need for skills-based, intensive, non-traditional education opportunities in additional areas. Few academic institutions offer advanced, hands-on training for research professionals looking to integrate in-demand methods into their biomedical research. SHARP trainings are designed for all career levels and sectors, including graduate students and postdoctoral scientists, government and corporate sector staff, and senior level research faculty and staff.

Trainings were initially offered in-person at the Columbia Mailman School. During the height of the COVID pandemic, all trainings were transitioned to livestream, virtual offerings. As of 2023, trainings are now offered in multiple formats, including in-person in New York City, livestream virtual, or hybrid (in-person and livestream virtual).

## 4 Assessment and results

The SHARP program began in 2017 with 105 participants in three sessions on two topics, and by 2022 had grown to 785 participants across 20 different offerings. From 2017 to 2022, 2,845 attendees from all 50 US states, 45 countries, and 580 unique organizations participated in 74 trainings. 13.1% of attendees (*n* = 373) participated in multiple trainings. SHARP serves a diverse participant base, encompassing all levels of professional seniority in a multitude of sectors (Table 2).

**Table 2:** Columbia University Mailman School of Public Health SHARP participant characteristics, 2017–2022.

**Race/Ethnicity**	***n***	Percentage of those responding^*^
American Indian/Alaska Native	3	0.2
Asian	398	26.4
Black	135	9.0
Hispanic	108	7.2
Native Hawaiian/Other Pacific Islander	5	0.3
White	677	45.0
Other	20	1.3
Selected Multiple Races	85	5.6
Prefer Not to Answer	74	4.9
Total number of respondents	1540	97.7^†^
**Professional role**	***n***	**Percentage of those responding**
Academic/Non-Profit Staff	212	11.7
Academic Faculty Member	472	25.9
Corporate/For-profit Staff	56	3.1
Government Staff	39	2.1
Postdoc/Trainee	636	31.6
Student	486	25.6
Total number of respondents	1788	63.9^†^

^*^Race/Ethnicity data collection started in 2021.

^†^Percentage of total participants responding at the time of registration.

At the completion of each workshop, participants were asked to respond to a short survey regarding their experience, impressions of the structure and content, and recommendations for future improvements. Surveys were closed and results tabulated after 14 days. With a post-training evaluation response rate of 65% (*n* = 1,841), 95% of attendees reported the training as having met or exceeded their expectations. 89% of attendees reported they would recommend the training to a colleague. Administrators have reported budgeting for SHARP workshops on new training grant proposals, especially for early career awards. Scholarship awards increased 62% from 2019 (when they were first available) to 2022, strongly indicating increasing interest. Enrollment in available seats across all programs has remained over 90% even as additional trainings and seats have been added, with 99% of seats filled in 2021. Multiple programs regularly reach capacity and have large waiting lists.

While a systematic evaluation of how participants later utilize the skills learned from SHARP courses would offer valuable insights into the long-term success of these workshops, such follow-up was beyond the scope of the initial workshop objectives.

## 5 Practical implications, objectives, and lessons learned

The SHARP program's value is providing training and education for professionals seeking to upskill and remain current in their fields. Through its intensive training programs, SHARP equips participants with the latest tools and techniques for addressing current and complex public health challenges. SHARP's concise format facilitates access to learners across public health and biomedicine, fostering collaboration and cross-disciplinary learning and broadening participants' understanding of public health issues and approaches. Through hands-on learning, mentorship, and exposure to new ideas and approaches, participants expand their knowledge, skills, perspectives, and professional networks, becoming more effective and confident public health professionals. SHARP's focus on emerging areas of research and practice empowers participants to tackle complex public health challenges and drive new discoveries and innovations.

Opportunities for future growth of short, intensive programs in public health are vast. As the field continues to evolve and new challenges emerge, the need for such programs will only increase. The SHARP program at Columbia University Mailman School of Public Health provides a template for addressing this need. Positive evaluations of SHARP demonstrate significant potential for further development and expansion in this educational area. This example can serve as a model for other institutions aiming to develop similar programs to meet the needs of public health professionals.

## 6 Acknowledgments of constraints

The SHARP program is self-funded. Attendees pay a participation fee that covers the costs of planning, instructor time, and infrastructure. The fee varies based on the real costs incurred in administering the workshop, which varies by workshop. Corporate partners are recruited to help offset costs and reduce the cost to participants but play no role in curriculum development. Any materials describing products or services that sponsors wish to share with participants are fully vetted by SHARP leadership and shared after completion of the program only if they are determined to be useful resources. Partnerships are disclosed on the SHARP website and in workshop materials provided to participants. Scholarships are available for eligible early-career candidates on the basis of scientific merit and financial need, with a particular focus on increasing participation of underrepresented groups and participants who would be unable or less likely to attend the training otherwise.

While we keep fees as low as possible, the true cost to run a high-quality, 2-day training averaged $25,000 at the Columbia University Mailman School of Public Health at the time of this manuscript preparation. This estimate included instructor and administrative preparation, outreach and attendee communication, nominal payments for all supporting individuals, and infrastructure (e.g., room costs, AV/IT, food, platforms, etc.). By their nature, these costs are variable and dependent on the location and timing of the training.

Feedback from participants indicated that part of the appeal of SHARP are the interactive nature and opportunities for networking. However, registration costs and travel for in-person trainings are financial barriers for attendance. To help overcome this obstacle and to maintain offerings during COVID, we introduced virtual livestreamed options scheduled for 7–8-h blocks during the typical US workday. Livestreamed options increase accessibility, but the currently available technology has many limitations for virtual attendees to feel similarly engaged as in-person attendees. While virtual trainings reduce participant costs, time zones are a challenge for synchronous workshops that are intended to be interactive and provide opportunities for networking.

We explored asynchronous formats, but feedback from instructors and attendees has not supported an offline model. Participants indicated that one of the reasons they preferred SHARP workshops to other educational opportunities was the opportunity to interact with instructors and other participants, which is not easily feasible in an asynchronous format.

While the post-training evaluation surveys provided snapshots of participants' impressions immediately after completing the workshops, we do not have data on the long-term effects on participants. However, as of 2022, over 13% of participants enrolled in multiple workshops, indicating they found the first workshop valuable enough to explore an additional topic area.

We foresee continued need for short, continually evolving trainings in research methodologies and professional development, but predicting specific topics to offer remains a challenge. Running trainings comes with uncertainties and risks, as the viability and success of a new training topic is unknown until the first iteration is complete. Success is only determined after substantial investment in time, effort, and funding by instructors and administrators. Additionally, the learning landscape rapidly changed in response to the pandemic. Travel costs and time away from family are viewed differently now that virtual offerings have increased and become more engaging. Virtual trainings offer improved accessibility but compete with other priorities in the usual work/home environment, unlike in-person events with dedicated learning time. It is unclear which training modality will prove most popular long term.

## Data Availability

The original contributions presented in the study are included in the article/supplementary material, further inquiries can be directed to the corresponding author.
